# Pressure gradient measurement in the coronary artery using 4D PC-MRI: towards noninvasive quantification of fractional flow reserve

**DOI:** 10.1186/1532-429X-16-S1-O55

**Published:** 2014-01-16

**Authors:** Zixin Deng, Zhaoyang Fan, Guoxi Xie, Yi He, Yutaka Natsuaki, Ning Jin, Xiaoming Bi, Jing An, Xin Liu, Zhaoqi Zhang, Zhanming Fan, Debiao Li

**Affiliations:** 1 Biomedical Imaging Research Institute, Cedars Sinai Medical Center, Los Angeles, California, USA; 2 Department of Bioenginnering, University of California, Los Angeles, Los Angeles, California, USA; 3 Shenzhen Institutes of Advanced Technology, Chinese Academy of Science, Shenzhen, China; 4 Department of Radiology, Anzhen Hospital, Beijing, China; 5 R&D, Siemens Healthcare, Los Angeles, California, USA

## Background

Fractional flow reserve (FFR) is an invasive procedure evaluating the functional significance of an intermediate stenosis by measurement of pressure drop across stenosis[1]. Noninvasive pressure measurement technique i.e. Phase-contrast (PC)-MRI has been studied in the cardiac chamber[2], aorta[3], and renal[4] arteries. The purpose of this study was to investigate the feasibility of pressure gradient quantification using 4DPC-MRI in the coronary arteries, which may allow for the derivation of FFR associated with stenosis.

## Methods

A 4D PC-MRI sequence with an acquisition window at the mid-diastole and end-expiration phase using ECG-triggering and navigator-gating to minimize motion-induced errors was implemented on a 3T system (MAGNETOM Verio, Siemens). The sequence measures the 4D flow velocity field through a cross-sectional 3D acquisition, in conjunction with the Navier-Stokes equations[2] to calculate the pressure gradient within the vessel segment of interest. A flow phantom study (gadolinium-doped water flow at a constant volume velocity of 250 mL/min in a silicone tubing of 4.8-mm ID) was first performed to determine the feasibility of the technique to detect changes in pressure difference (ΔP) at six different stenosis cases: 0, 22%, 34%, 44%, 60%, 64% with appropriate combinations of VENCs in z (45, 60, ..., 200 cm/s) and x, y (20, 30, ..., 80 cm/s) directions. The sequence was then tested in 3 healthy male volunteers using a VENC of 90z40x40y on the left main, LCX and 60z30x30y on the proximal LAD, respectively. Imaging parameters for human studies were: spatial resolution = 0.78 × 0.78 × 2.00 mm^3^, flip angle = 15°, cardiac phase = 2-3 (77 ms/phase) coinciding with the quiescent period, scan time = 11-18 mins.

## Results

**Figure 1:**
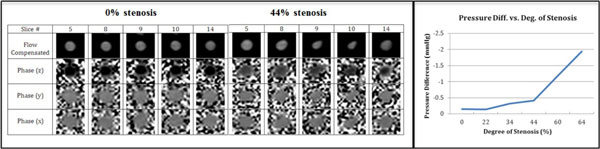
**Example images for flow phantom at 0% and 44% stenosis and pressure difference curve in phantom showing the pressure difference between the most stenotic slice and the reference (2nd) slice increased with the degree of stenosis**.

**Figure 2:**
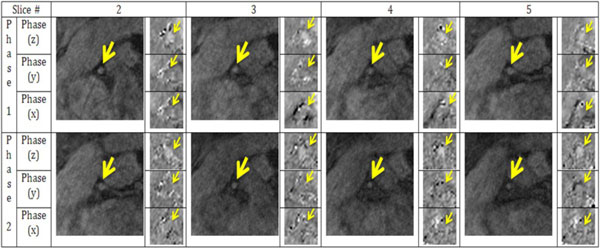
**Example images of flow compensated (reference) and phase difference (x, y, z) images for two cardiac phases in a healthy volunteer**.

Phantom studies: 16 contiguous slices were acquired spanning the stenosis area. ΔP between the most stenotic slice and the reference (2nd) slice increased with the stenosis degree, as illustrated in Figure 1. Volunteer studies: 6 contiguous slices were acquired per volunteer. Figure 2 illustrates the flow compensated (reference) and phase difference (x, y, z) images of one volunteer from 2 successive cardiac phases during the mid-diastole, where the yellow arrows are pointing at the cross-sections of the coronary artery. Cardiac phases in the z- and x, y-direction differed by 6-15 cm/s and 0.5-5 cm/s, respectively. ΔP values between slices 2 and 5 were 0.1646, 0.1407 and 0.2259 mmHg in the 3 volunteers, respectively.

## Conclusions

The preliminary results have suggested that quantification of pressure gradient in the coronary artery is feasible. As expected, healthy volunteers showed a near zero pressure gradient across the coronary arteries. Animal and clinical validations on real coronary stenosis are currently underway. Further technical improvements such as temporal/spatial resolution are warranted.

## Funding

NHLBI HL38698, NIBIB EB002623.
